# Targeting vaccinations for the licensed dengue vaccine: Considerations for serosurvey design

**DOI:** 10.1371/journal.pone.0199450

**Published:** 2018-06-26

**Authors:** Natsuko Imai, Neil M. Ferguson

**Affiliations:** MRC Centre for Global Infectious Disease Analysis, Department of Infectious Disease Epidemiology, Imperial College London, London, United Kingdom; Public Health England, UNITED KINGDOM

## Abstract

**Background:**

The CYD-TDV vaccine was unusual in that the recommended target population for vaccination was originally defined not only by age, but also by transmission setting as defined by seroprevalence. WHO originally recommended countries consider vaccination against dengue with CYD-TDV vaccine in geographic settings only where prior infection with any dengue serotype, as measured by seroprevalence, was >170% in the target age group. Vaccine was not recommended in settings where seroprevalence was <50%. Test-and-vaccinate strategies suggested following new analysis by Sanofi will still require age-stratified seroprevalence surveys to optimise age-group targeting. Here we address considerations for serosurvey design in the context of vaccination program planning.

**Methods:**

To explore how the design of seroprevalence surveys affects estimates of transmission intensity, 100 age-specific seroprevalence surveys were simulated using a beta-binomial distribution and a simple catalytic model for different combinations of age-range, survey size, transmission setting, and test sensitivity/specificity. We then used a Metropolis-Hastings Markov Chain Monte-Carlo algorithm to estimate the force of infection from each simulated dataset.

**Results:**

Sampling from a wide age-range led to more accurate estimates than merely increasing sample size in a narrow age-range. This finding was consistent across all transmission settings. The optimum test sensitivity and specificity given an imperfect test differed by setting with high sensitivity being important in high transmission settings and high specificity important in low transmission settings.

**Conclusions:**

When assessing vaccination suitability by seroprevalence surveys, countries should ensure an appropriate age-range is sampled, considering epidemiological evidence about the local burden of disease.

## Introduction

The total incidence of dengue cases has increased 30-fold in the past 50 years. Dengue infection was the cause of an estimated 1·14 million (0·73 million– 1·98 million) disability-adjusted life-years (DALYs) in 2013 [1]. Consequently, dengue is now the most important mosquito-borne viral infection globally, and with 40% of the world’s population at risk of infection, dengue imposes a significant public health burden [2,3].

The first dengue vaccine—the Sanofi CYD-TDV vaccine, has now been licensed by several dengue-endemic countries for use in 9–45 years and 9–60 year olds; including in Brazil, El Salvador, Paraguay [4], Costa Rica [5], Mexico [6], and the Philippines [7]. Results from large-scale phase III trials conducted in 2–14 year olds in 5 countries in Asia showed a efficacy of 67% (95% CI: 50%–79%) against hospitalised disease in 25 months of follow-up after the first dose of vaccination [8]. The CYD15 trial conducted in 9–16 year olds in 5 countries in Latin America showed a vaccine efficacy of 80% (95% CI: 70%–88%) against any hospitalised dengue infection after one year of active follow-up following the last dose of vaccination [9]. Vaccine efficacy in both trials varied by infecting serotype, age, and baseline serostatus [8–10]. However, in vaccinated children aged 2–5 years in Asia, there was a statistically significant increased risk of hospitalised dengue compared with controls in the second year of (passive-surveillance based) follow-up after the last dose of vaccine [9]. This pattern continued in the third year of follow-up and contributed to the decision by the manufacturer to only seek licensure of the vaccine for use in children aged 9 years and above [11].

These trial results led to the WHO Strategic Advisory Group of Experts (SAGE) recommending the introduction of the vaccine only in high endemic regions (>70% seroprevalence in the target age group, 9 years and above). Vaccination was not recommended where seroprevalence is <50% in the target age group [12]. Following these recommendations, there was considerable debate about the public health impact and safety profile of CYD-TDV within the scientific community [13–19]. More recently, new analysis of the CYD-TDV data from Sanofi demonstrated higher risk of severe dengue amongst individuals seronegative at baseline for any age. This has led to Sanofi asking regulators to update their product label to reflect these new findings and WHO to release new interim guidelines recommending that only individuals with prior evidence of dengue exposure receive the vaccine [20,21]. Considering these new findings, age-stratified seroprevalence surveys will still be needed to optimise age-group targeting for test-and-vaccinate vaccination programmes. Thus, accurate assessment of the baseline seroprevalence of a population is essential prior to any small or large-scale implementation of the vaccine.

**Fig 1:**
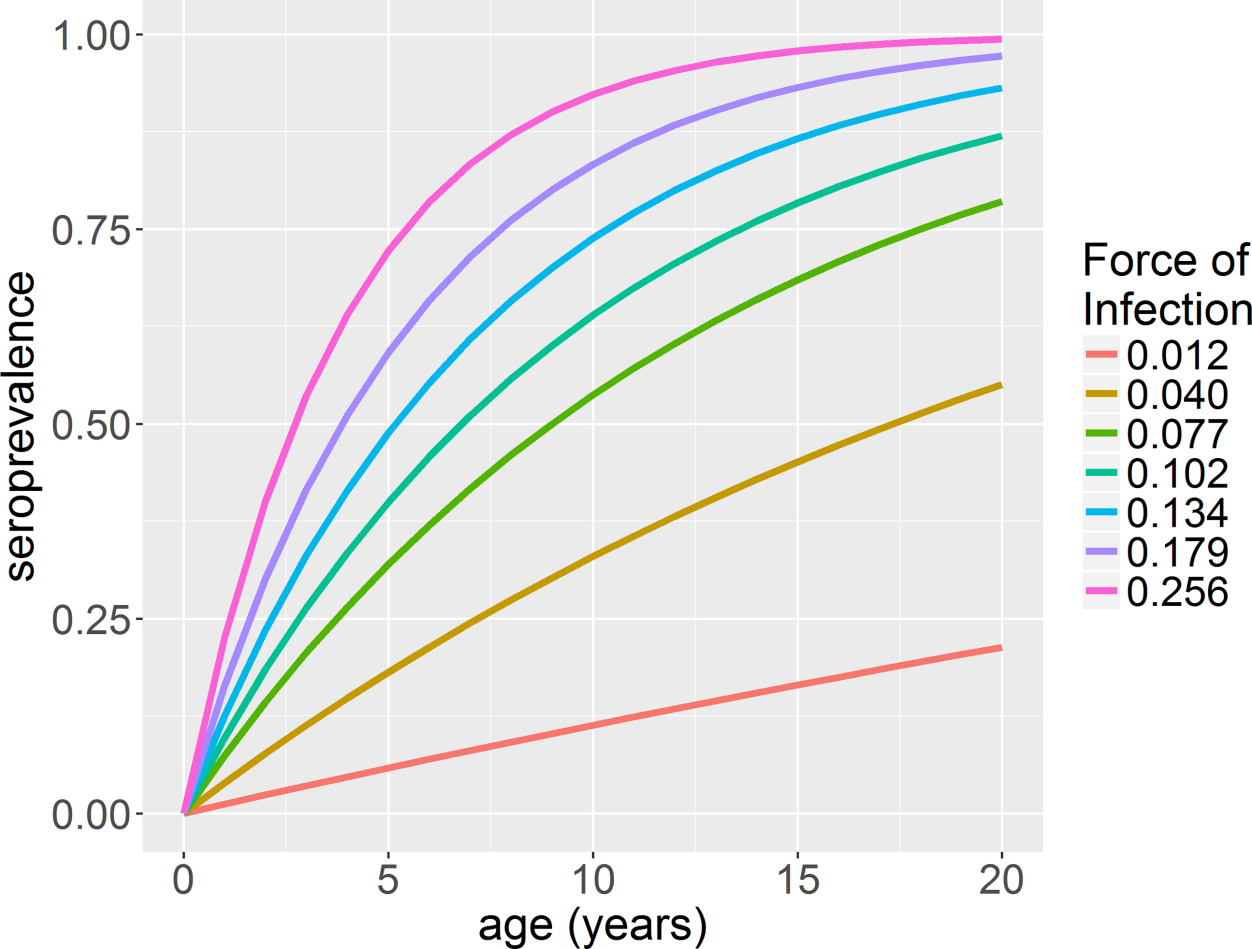
Change in seroprevalence with age at different transmission intensities. Transmission intensity values are given at the end of each seroprevalence curve.

Age-specific seroprevalence surveys can show considerable fluctuations with age due to the epidemic nature of dengue transmission as well as sampling variability. Therefore, the observed seroprevalence in a single age group may not be representative of the long-term average seroprevalence of the target age group (Figure K in S1 Text). A more accurate measure of dengue endemicity is the average transmission intensity, as quantified by the force of infection, λ. This is defined as the per capita rate that susceptible individuals acquire infection [22] and since it takes into account population susceptibility, it can be used to compare the intensity of dengue transmission in different populations [23]. The force of infection for dengue can be estimated from non-serotype and serotype-specific age-stratified seroprevalence data [24]. However, accurate estimation requires seroprevalence data from a broad sample age range, due to the highly non-linear relationship between age-specific seroprevalence and force of infection (Fig 1).

In the context of the SAGE recommendations for the CYD-TDV vaccine, here we present considerations when designing seroprevalence surveys for targeting vaccination such as; the age-range from which we should sample, the survey size, and how the sensitivity and specificity of the assay may affect our estimates.

## Methods

### Model

Assuming that seroprevalence surveys are conducted using non-serotype specific assays such as the IgG enzyme-linked immunosorbent assays (ELISA), we assumed that the relationship between the seroprevalence and age is given by a simple catalytic model [22,23]:

$$z_{a}=1-\exp[-\lambda a].$$ (1)

Where *z*_*a*_ is the seroprevalence in age group *a*, *λ* is the force of infection, and *a* is age in years. The model assumes a constant force of infection over time and generally describes the results of cross-sectional IgG surveys well [24].

### Simulation and estimation procedure

We considered 11 potential age ranges to test and 5 total survey sizes. We assumed that individuals were distributed uniformly across the age groups, i.e. for a total survey size of 2000 and an age range of 0–20 year olds; 96 individuals are tested in each year of age. We also considered 7 different transmission settings by varying the force of infection to give a true seroprevalence in 9-year olds (the target age group) which ranged between 10–90% (P10 to P90). Finally, we looked at different test sensitivities and specificities in the range 90–100%. Table 1 summarises the combinations tested.

**Table 1:** Summary of different scenarios used for simulated seroprevalence surveys. Each variable is varied independently. The last three scenarios for the test sensitivity/specificity column represent reported performance of commercial tests.

Age Range of survey (years)	Total Survey Size	Transmission Setting: % seroprevalence at age 9 (*λ*)	Test sensitivity / specificity
0–20			
5–20		10 (0.012)	100% / 100%
10–20	2000	30 (0.040)	90% / 90%
15–20	1500	50 (0.077)	95% / 95%
5–10	1000	60 (0.102)	90% / 99%
5–15	750	70 (0.134)	99% / 90%
5–18	500	80 (0.179)	96.3% / 91.4% *[25]
9–12		90 (0.256)	96% / 93% ^[26]
9–15			98.8% / 99.2%¯[27]
9–18			

*PanBio IgG assay.

^Focus Diagnostics.

¯Standard Diagnostics Inc.

For every combination of age range, survey size, transmission setting, and test sensitivity-specificity, we simulated 100 seroprevalence surveys. We assumed that the probability of an individual in age group *a* being seropositive was beta-binomially (BB) distributed (2):

$$p_{a}∼BB\left(N_{a},z_{a},\gamma \right),$$ (2)

where *N*_*a*_ is the number of individuals in age group *a*, *z*_*a*_ is the proportion of the age group seropositive given by Eq (1), and *γ* is over-dispersion which represents the underlying variation in transmission intensity within the sampled population (the smaller the value the larger the over-dispersion). Therefore *p*_*a*_ takes into account the overdispersion, while *z*_*a*_ is dependent solely on the force of infection and age. Over-dispersion was fixed at *γ* = 25 (average value estimated from fitting model (1) with the beta-binomial parameterisation specified in Eq (4) to 28 seroprevalence surveys from 13 countries referenced in Imai *et al*. [24]).

The sensitivity and specificity determines the probability that an individual in age group *a* will test positive, *P*(*T*_*a*_^+^):

$$P\left(T_{a}^{+}\right)=p_{a}S_{e}+\left(1-p_{a}\right)\left(1-S_{p}\right)$$ (3)

**Fig 2:**
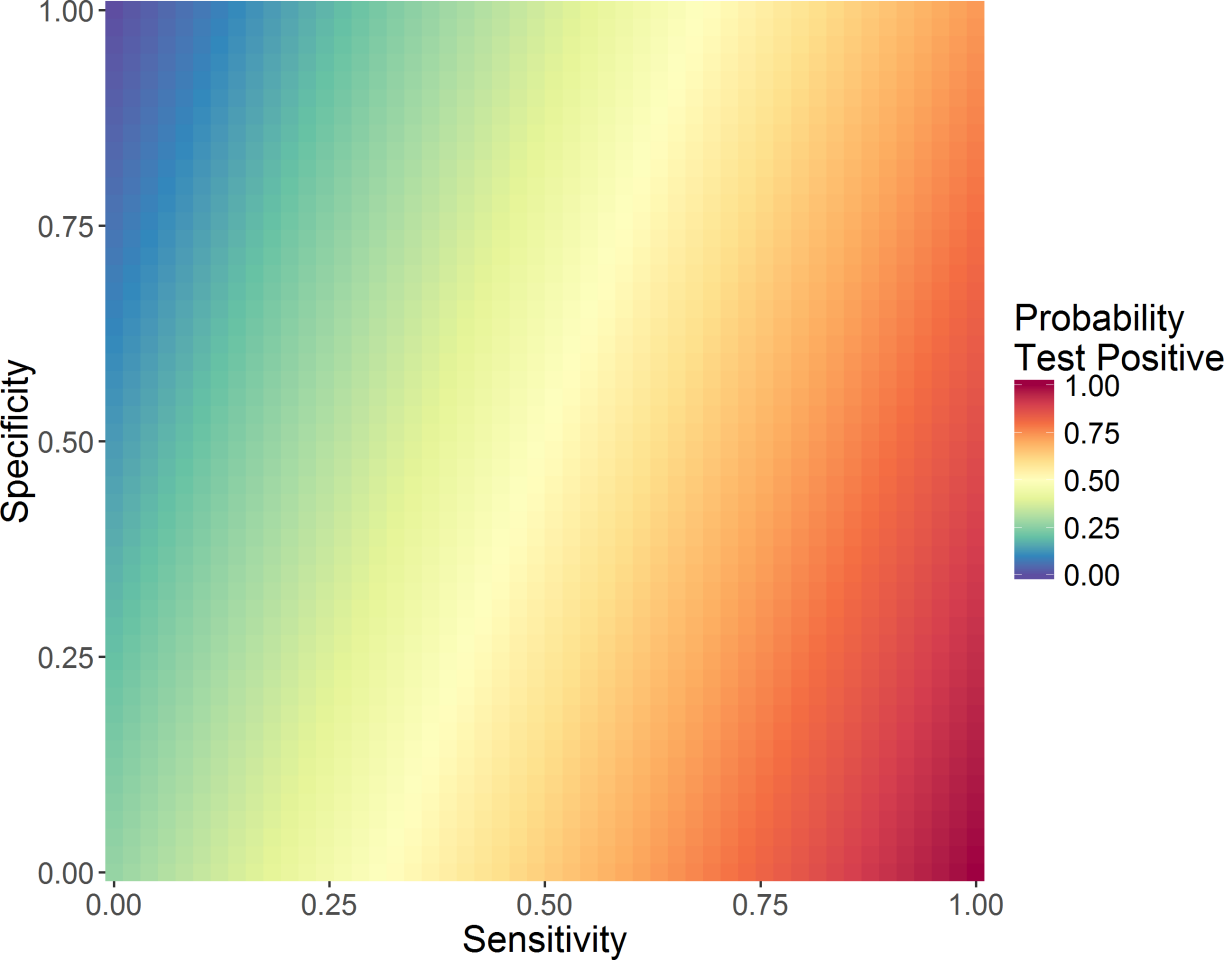
Probability of 9-year olds testing positive [P(T_a_^+^)] for different test sensitivity and specificities, for a true probability of being seropositive of 0.7.

Here *p*_*a*_ is the probability an individual aged *a* is seropositive (2) and *S*_*e*_ and *S*_*p*_ are the sensitivity and specificity of the IgG test, respectively. Thus, with a perfect test (100% sensitivity and specificity), *P*(*T*_*a*_^+^) = *p*_*a*_. Fig 2 shows the probability of testing positive at different sensitivity and specificities given that the baseline probability of being seropositive is 0·7.

For comparison purposes, the same analysis was undertaken without incorporating over-dispersion in serosurvey results (i.e. sampling from a binomial rather than a beta-binomial distribution). For full details see S1 Text.

The force of infection (*λ*) and overdispersion (*γ*) was then estimated from each simulated dataset (100 estimates per parameter combination) using a Metropolis-Hasting Markov Chain Monte-Carlo (MH-MCMC) algorithm with a beta-binomial likelihood (4) and uniform priors. The model likelihood is given by:

$$L=\prod \frac{B\left(N_{a}-X_{a}+\gamma \left[1-P\left(T_{a}{}^{+}\right)\right],X+\gamma P\left(T_{a}{}^{+}\right)\right)}{B\left(\gamma \left[1-P\left(T_{a}{}^{+}\right)\right],\gamma P\left(T_{a}{}^{+}\right)\right)}$$ (4)

And the log likelihood by:

$$LnL=\sum_{a}\left[\begin{array}{l} \ln\left\{B\left(N_{a}-X_{a}+\gamma \left[1-P\left(T_{a}{}^{+}\right)\right],X+\gamma P\left(T_{a}{}^{+}\right)\right)\right\}-\\ \ln\left\{B\left(\gamma \left[1-P\left(T_{a}{}^{+}\right)\right],\gamma P\left(T_{a}{}^{+}\right)\right)\right\} \end{array}\right]$$ (5)

Here *X*_*a*_ is the number of individuals testing seropositive in age group *a*, *N*_*a*_ is the total number of individuals tested in age group *a*, and *P*(*T*_*a*_^+^) is the probability that an individual in age group *a* tests positive as defined above (3). The model was fitted using the R programming language (version 3.1.0) [28]. For details on the binomial estimation procedure see S1 Text.

We computed the mean, standard deviation and coefficient of variation (standard deviation divided by mean) of the mean posterior estimates of the force of infection for each combination of age range, survey size, transmission setting, test sensitivity and test specificity.

## Results

**Fig 3:**
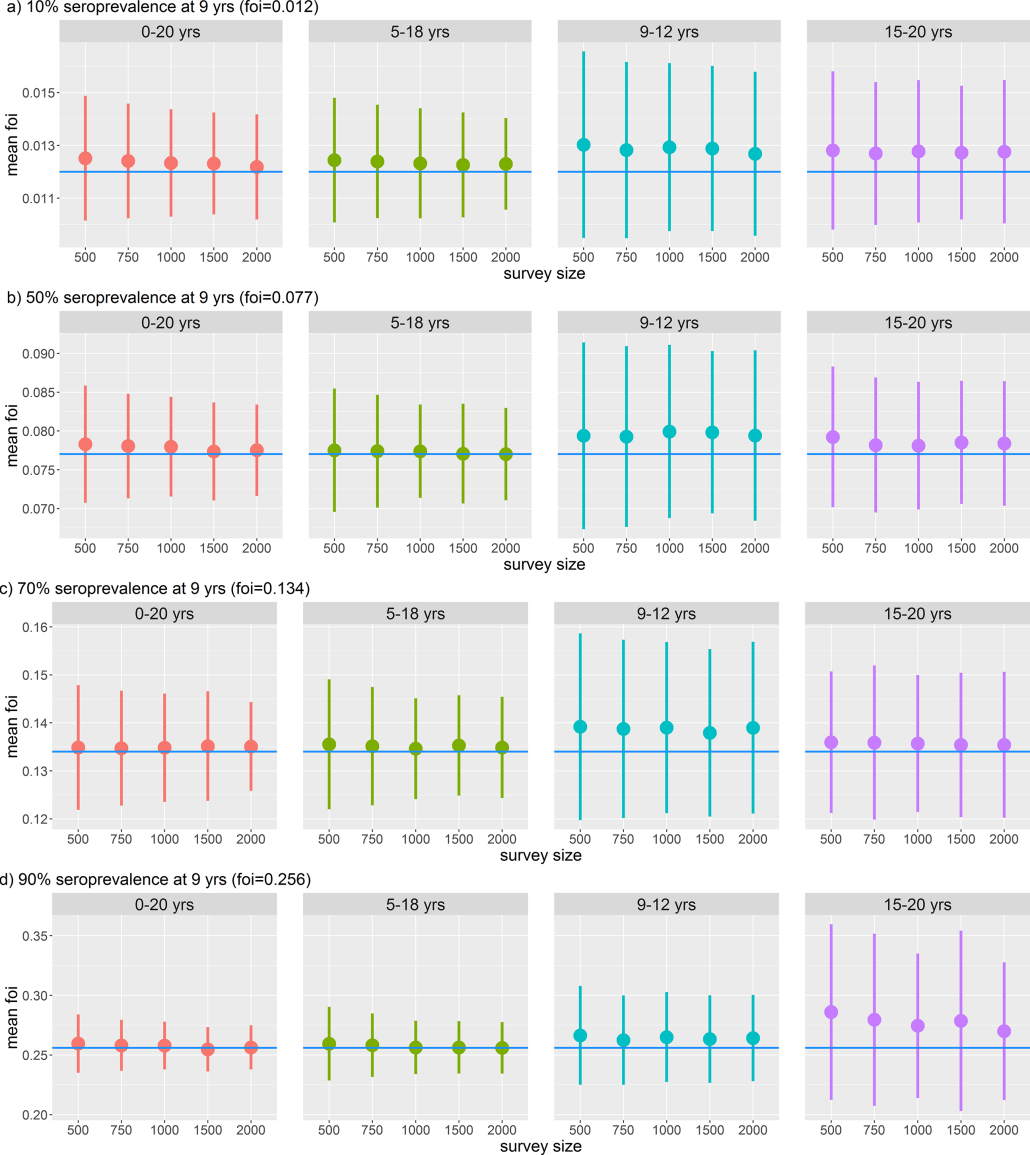
Dengue force of infection estimated from different age ranges and survey sizes at different transmission settings assuming a beta-binomial distribution and a perfect test is used (100% sensitivity and specificity). A) Very low transmission setting (10% seroprevalence at 9 years), B) medium transmission setting (50% seroprevalence at 9 years), C) high transmission setting (70% seroprevalence at 9 years), and D) very high transmission setting (90% seroprevalence at 9 years). The point is the mean of the mean posterior estimates from 100 simulations and the line the standard deviation. The blue line shows the true value of the force of infection.

With a perfect (100% sensitivity and specificity) test, Fig 3 shows that biases in the estimates of force of infection from simulated surveys were small when wide (0–20 years or 5–18 years) age ranges were sampled, but consistently over-estimated for surveys which covered a narrower age range (9–12 of 15–20). In very low transmission settings (10% seroprevalence at 9 years), even with the widest age range (0–20 years), the force of infection was slightly overestimated (Fig 3A). In very high transmission settings (90% seroprevalence at 9 years), restricting sampling to older children led to the force of infection being over-estimated (Fig 3D).

As expected, the precision of the estimate increased with survey size for all transmission settings and age ranges examined (smaller standard deviation around the central estimate) (Fig 3). However, the width of the age range sampled had a greater impact on the precision of the estimate than sample size. With a narrow age range (e.g. 9–12 years), although the central estimate was close to the true value, uncertainty around the estimate was large and could not be substantially reduced by increasing survey size. This finding was consistent across all transmission settings (Fig 3A–3D) but was not as marked when we did not account for over-dispersion (Figure B in S1 Text).

We found it was always optimal to sample from the widest age range (0–20 years, followed by 5–18 years). However, if sampling such a wide range is impractical, in low transmission settings (FOI: 0·012, expected seroprevalence at age 9 = 10%) it is most important to sample from older age groups since the difference in seroprevalence amongst young children (<9 years) is small. Fig 3A shows that sampling 15–20 year olds gives a more precise estimate than sampling from 9–12 year olds in this setting. Conversely, in high transmission settings (FOI: 0·256, expected seroprevalence at age 9 = 90%) it is important to sample from younger age groups since differences in seroprevalence among older ages will be small.

We found that there was no substantial gain in precision of our estimates above a survey size of 1000. As expected, the larger the survey size, the more precise our estimates were (Fig 3).

**Fig 4:**
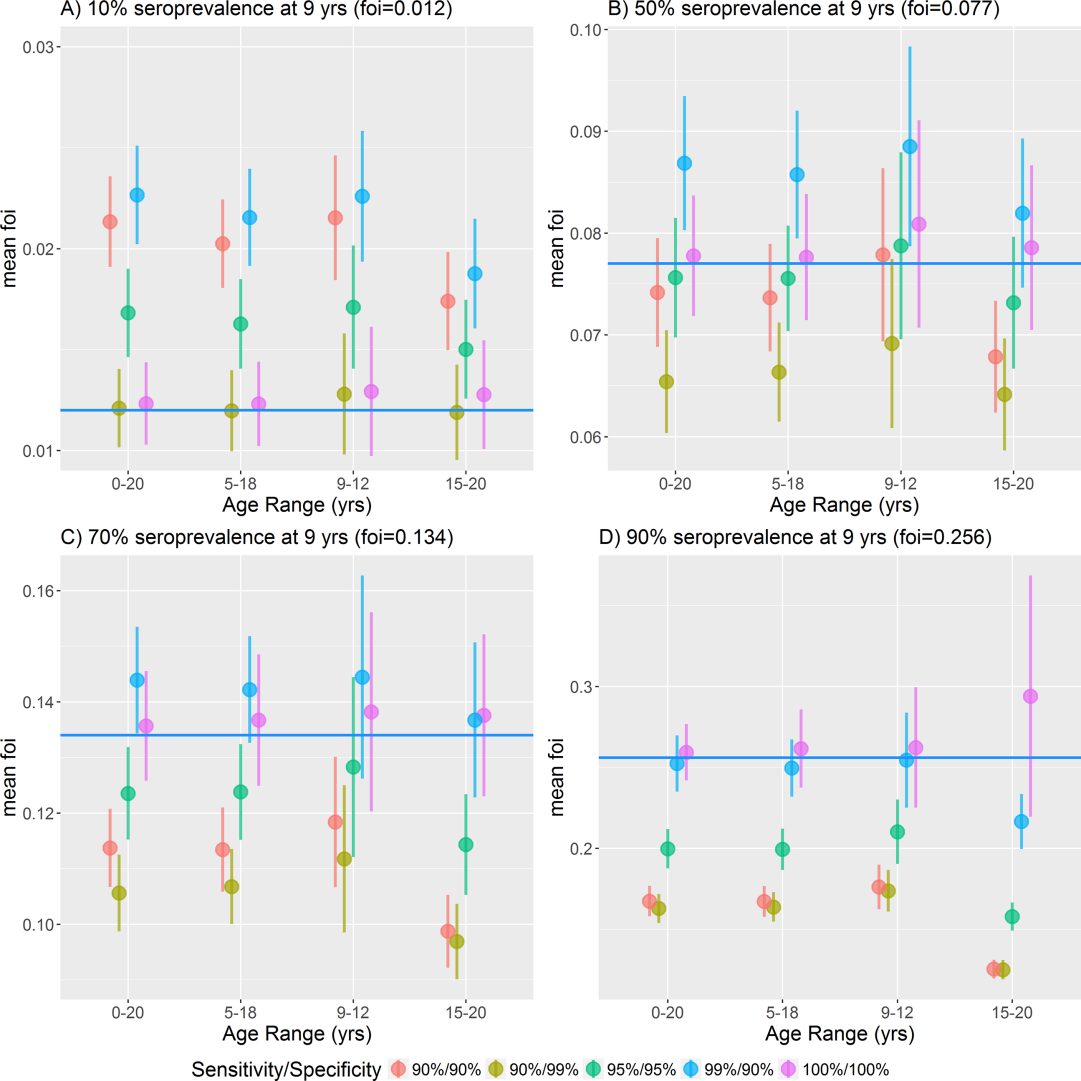
Dengue force of infection estimated at different transmission intensities, from a range of ages and test sensitivities and specificities, fixed survey size of 1000. A) Very low transmission 10% seroprevalence at age 9, B) medium transmission 50% seroprevalence at age 9, C) high transmission 70% seroprevalence at age 9, D) very high transmission 90% seroprevalence at age 9. The point shows the mean of the 100 mean posterior distribution of the force of infection, the bar the standard deviation, and the horizontal blue line shows the true force of infection.

We examined the effect of different test sensitivities and specificities on the ability to accurately estimate the force of infection. As expected, a hypothetical perfect test (100% sensitivity and specificity) performed best. For imperfect tests, there was a trade-off between sensitivity and specificity depending on the transmission setting. In low transmission settings, having an assay with a high specificity (percentage of seronegative individuals correctly identified as negative) was more important than sensitivity (percentage of seropositive individuals correctly identified as positive) for reliable estimation of the force of infection (Fig 4A). Conversely in high transmission settings (Fig 4C and 4D) having an assay with a high sensitivity was more important than specificity. In medium transmission settings (P9 = 50%) having an assay with balanced sensitivity and specificity was most beneficial.

**Fig 5:**
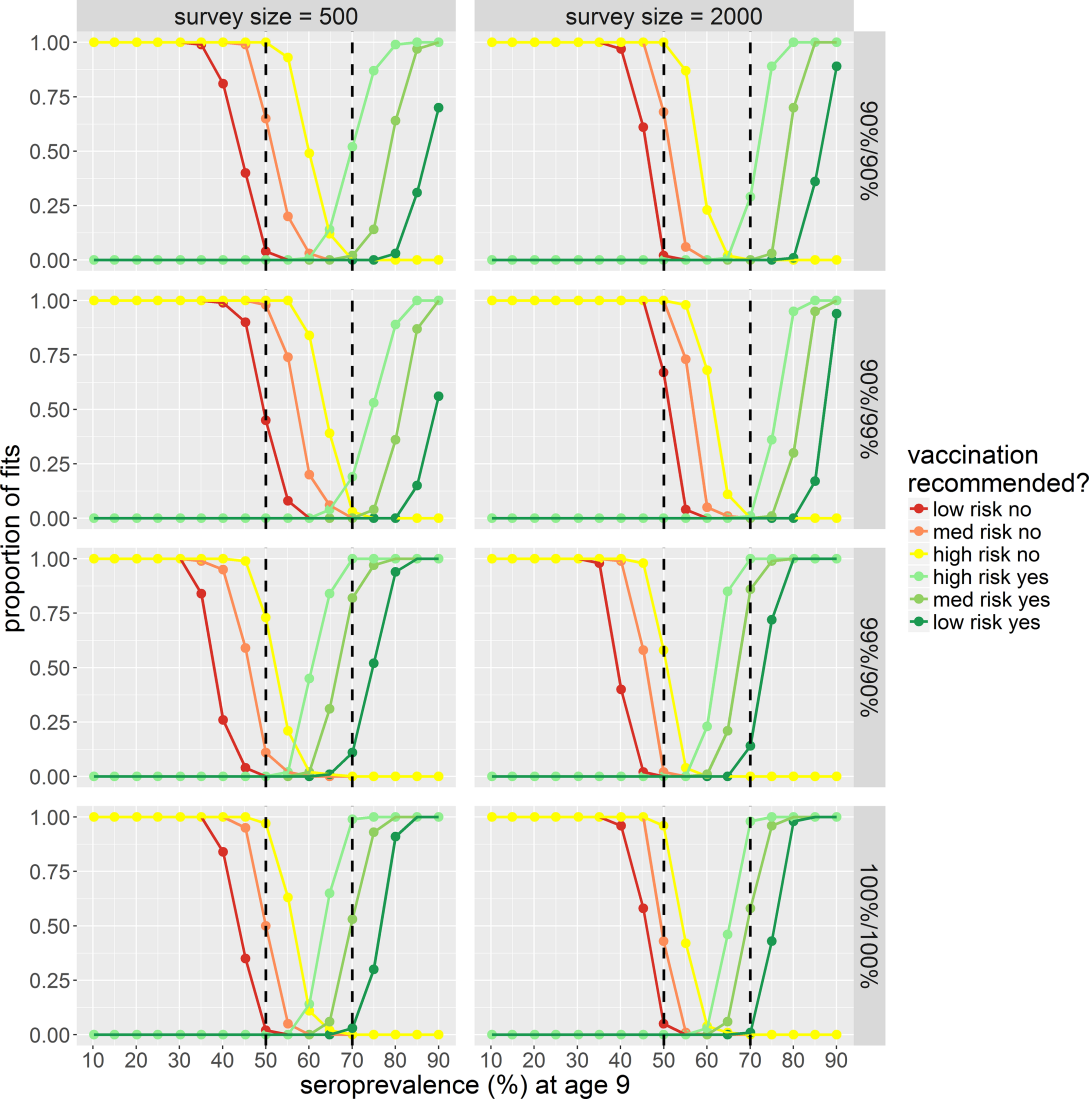
Proportion of model fits (of 100) which result in the correct vaccination recommendation for a range of transmission settings defined by seroprevalence in 9 year olds (low: P9 = 10%, to high: P9 = 90%). Rows show results for different test sensitivities and specificities (Se%/Sp%). Columns for survey sizes of 500 and 2000, estimated from a fixed age range of 0–20 year olds. The black dashed lines represent seroprevalence thresholds of 50% and 70%. Red = vaccination is not recommended, green = vaccination is recommended. Low risk no = upper 95% CrI < 50%, med risk no = central estimate <50%, high risk no = upper 95% CrI < 50%, high risk yes = upper 95% CrI above 70%, med risk yes = central estimate >70%, low risk yes = lower 95% CrI >70%. CrI = credible interval.

This result is also reflected in Fig 5 which shows the proportion of fits making the correct recommendation regarding vaccination for a range of transmission settings (defined by seroprevalence in 9-year olds, P9 = 10% to 90%). We define the vaccination recommendation risk as the proportion of fits (of 100 simulations) where the central estimate, upper or lower 95% credible interval (CrI) of the estimated seroprevalence at age 9 (P9) falls above or below the 50% or 70% seroprevalence thresholds. For ruling out vaccination: i) lowest risk criterion–the upper 95% CrI is below 50% seroprevalence; ii) medium risk criterion–central estimate is below 50%; and iii) highest risk criterion–lower 95% CrI is below 50%. For recommending vaccination: i) lowest risk criterion–lower 95% CrI is above 70% seroprevalence; ii) medium risk criterion–central estimate is above 70%; and highest risk criterion–upper 95% CrI above 70%.

The proportion of fits correctly recommending against vaccination when the baseline seroprevalence was <50% decreased when using a lower specificity assay (Fig 5). Conversely the proportion of fits correctly recommending vaccination when the baseline seroprevalence was >70% decreased when using a low sensitivity assay. Unsurprising, of the commercially available assays, the assay with the highest sensitivity and specificity–the IgG ELISA from Standard Diagnostics Inc. (*S*_*e*_ = 98.8%, *S*_*p*_ = 99.2%), performed the best (Figures G and H in S1 Text).

## Discussion

The results from these simulations highlight several key considerations in the design of serosurveys to assess dengue vaccine suitability. For the target age range, we found that having a wide age range from which to survey was more important to accurately assess the force of infection than a having a large survey size with a narrow age range (Fig 3). There was no substantial gain in precision above sample sizes of 1000 individuals. In high transmission settings (P9>70%) younger children under 9 years of age were most important to include, while in low transmission settings (P9<30%) older aged children and even adults need to be sampled. For differences due to diagnostic sensitivity and specificity, we found that in low transmission settings, a test with a high specificity was favoured over sensitivity (Fig 4A and 4B). Using a low specificity assay would increase the number of false positive results, and in a low transmission setting increases the probability of overestimating the true seroprevalence. Conversely in high transmission settings, high sensitivity was more important than specificity (Fig 4B and 4C). Using a low sensitivity assay would increase the number of false negative results and in this setting would increase the probability of underestimating the true seroprevalence [29,30]. In medium transmission settings, having a balanced sensitivity and specificity was most important. Test sensitivity and specificity had a substantial impact on the accuracy of results in informing potential decisions about whether to introduce dengue vaccination according to the original WHO guidelines [31]. Seroprevalence estimates can be adjusted if test sensitivity and specificity are known. However, estimates have not been corrected here as with the increasing body of evidence concerning safety associated with the CYD-TDV vaccine, we felt seroprevalence estimates should be conservative.

CYD-TDV was originally licensed for use in individuals aged 9–45 years or 9–60 years depending on the country. Our finding that it is more important to sample from a wider age range (e.g. 5–18 years) than have a large survey in a narrow age range (e.g. 9–12 years), as well as the importance of sampling younger children in high transmission settings is important since children <9 years old are not eligible to receive the vaccine and are thus not an obvious target population in serosurveys. Conversely, in very low transmission settings, even with the widest age range of 0–20 years the transmission intensity was slightly overestimated. This slight overestimation is observed in very low to high transmission settings (P9 = 10, 50, and 70%) (Fig 3). This is a result of whether, for each transmission setting, the age range sampled from has sufficient differences in the seroprevalence to accurately estimate the force of infection. For example, in very low transmission settings (P9 = 10%), the seroprevalence curve will be flat, and there is not enough of a difference in seroprevalence between 0 years and 20 years. Conversely, in very high transmission settings (P9 = 90%), the change in seroprevalence within the target age group (0–20 years) is sufficient to accurately estimate the force of infection (Fig 3D panel 0–20 years). This suggests that older adults, in some cases above 45 years of age may also need to be sampled. This may be the case in countries (e.g. Singapore) where the seroprevalence in school-aged children is low [32–34]. In Singapore CYD-TDV has been licensed for private use only (12 years and above) with guidelines suggesting patients are tested to determine past infection status before vaccination [35].

If CYD-TDV or any of the dengue vaccines in development are to be introduced as a public programme, possibly with revised seroprevalence or testing thresholds, it is likely that vaccination will be rolled out at the sub-national or state level. However, dengue transmission intensity is geographically heterogenous even at fine spatial scales [36,37] and the force of infection can differ substantially between sub-districts of the same administrative region [38]. Therefore, it would be beneficial to conduct multiple smaller surveys (of sufficient size) to capture the geographic heterogeneity within a region rather than to conduct one large survey in e.g. the state capital [39].

There are currently no external independent standardisation protocols for dengue serological assays, and evaluations have focused on IgM assays for the diagnosis of acute dengue infection rather than for serological surveys [40–42]. As dengue serosurveys become more common with the introduction of public vaccination programmes, it will be important to have standardised serological assays.

Dengue antibodies are cross-reactive with other flaviviruses including Yellow Fever (YF), Japanese encephalitis (JE), West Nile Virus, and Zika [43–45], implying care must be taken when interpreting the results of serosurveys in areas with co-circulation of other flaviviruses, or routine JE or YF vaccination. With the emergence of Zika in South America and the large epidemic observed in 2015–2016, cross-reactivity may be of particular concern since there are currently no commercially available diagnostics or internationally recognised standards for serologically distinguishing past exposure to Zika and dengue [46]. In the context of conducting serosurveys to inform dengue vaccination, WHO recommend that a subset of samples is re-tested using neutralisation tests such as plaque-reduction neutralisation tests (PRNTs) as they do not detect the same cross-reactivity. Such validations will allow IgG ELISA cut-offs to be calibrated to the local epidemiological context. Additionally, having such baseline measurements may be important when assessing vaccine efficacy at a later date as this can depend on baseline serostatus [8–10]. Baseline age-stratified seroprevalence data will also allow comparison of the force of infection in the population before and after vaccination.

Our simple catalytic model assumes a constant force of infection in both age and time. While it is not possible to assess both time and age-dependent changes in transmission intensity from a single cross-sectional survey, age-dependent changes can be estimated by assuming transmission is constant over time and vice-versa [47–49]. Dengue transmission can vary year-to-year due to the cyclical nature of transmission and catalytic models have been applied to age-stratified dengue seroprevalence data to estimate time-varying force of infection [38,50–52]. However, estimates derived from cross-sectional IgG surveys assuming a constant force of infection still give a good estimate of the average transmission intensity over time within a population [24,51,52]. Since the dengue vaccine is intended for highly endemic areas, this should be sufficient to assess vaccination suitability. Additionally, although there may be serotype-specific differences in transmission intensity, non-serotype specific data can yield an estimate of the average total force of infection across all serotypes consistent with the sum of serotype-specific forces of infection derived from PRNT data [24].

Our main results did not change depending on whether we took overdispersion, which represents the underlying variation in transmission intensity within the sampled population, into account. However, the importance of also sampling from older or younger children in lower and higher transmission settings was not as marked when using a binomial model with no overdispersion (S1 Text). We found that assuming greater overdispersion makes it increasingly difficult to estimate transmission intensity accurately (Figure I in S1 Text). The degree of overdispersion is likely to differ by setting.

Since conducting serosurveys is resource-intensive and are often only conducted once, or every few years at most, it would be advantageous to be able to derive reliable force of infection estimates from routine surveillance data to supplement results from serosurveys. We have previously shown that dengue transmission intensity can be inferred from the age-distribution of notified dengue cases [53]. Since surveillance data are collected continuously, they could provide more detail of how transmission changes over a smaller timescale. However, additional age-stratified seroprevalence and incidence data which are matched in time and space are required to fully validate this approach of using surveillance data as a proxy for serological data.

The original WHO recommendations for CYD-TDV specified that countries only consider introduction of CYD-TDV in areas with >70% seroprevalence in the target age group [31]. However, seroprevalence in a single narrow age group can show major fluctuations from year to year due to the epidemic nature of dengue transmission and sampling variability [54]. Here we have shown that a more accurate measure of dengue endemicity is the force of infection (*λ*) which can be estimated from such serosurveys. Force of infection estimates can incorporate data from a much wider age group than the target age group for vaccination and can then be used to better estimate the long-term average seroprevalence at any given age. Such data would provide gold-standard data on dengue transmission against which routine dengue surveillance data could be compared. With new analysis from Sanofi prompting a change in product label and WHO to release interim guidelines recommending vaccination only in individuals with evidence of prior exposure, it remains to be seen whether seroprevalence thresholds at the population level could still be used to target CYD-TDV. However, it is likely that a test and vaccinate strategy may be adopted in some high burden settings where age-stratified serosurveys will still be needed to optimise age-group targeting for such vaccination programmes.

Although these results are presented in the context of the CYD-TDV dengue vaccine, phase II interim analysis of the Takeda TDV vaccine has also shown lower geometric mean titres (GMT) of neutralising dengue antibodies to DENV-1 to -4 in those seronegative at baseline compared to seropositive at baseline [55]. Therefore, it is possible that similar recommendations for targeting vaccination may be made for future dengue vaccines. Finally, the considerations for serosurvey design highlighted here can be applied not just to dengue but more broadly to seroprevalence surveys in general.

## Supporting information

S1 Text Supporting information. (DOCX)
