# Supplementary material for: Targeting vaccinations for the licensed dengue vaccine: Considerations for serosurvey design
Source: PLoS One. 2018 Jun 26;13(6):e0199450. doi: 10.1371/journal.pone.0199450 (PMC6019750; doi:10.1371/journal.pone.0199450)
Supplement: S1 Text — (DOCX) [file pone.0199450.s001.docx]

**Title: Targeting Vaccinations for the Licensed Dengue Vaccine: Considerations for Serosurvey Design Supplementary Information**

**Authors:** Natsuko Imai^1*^, Neil M Ferguson^1^

**Affiliations:** 1. MRC Centre for Global Infectious Disease Analysis, Department of Infectious Disease Epidemiology, Imperial College London, London, UK.

[*n.imai@imperial.ac.uk](mailto:*n.imai@imperial.ac.uk)

**Methods**

Model

Assuming that seroprevalence surveys are conducted using non-serotype specific assays such as the IgG enzyme-linked immunosorbent assays (ELISA), we assumed that the relationship between the seroprevalence and age is given by a simple catalytic model [1,2]:

| $z_{a}=1-\exp[-\lambda a]$. |  | (1) |
| --- | --- | --- |

Where $z_{a}$ is the seroprevalence in age group $a$, $\lambda$ is the force of infection or transmission intensity, and $a$ is age in years. The model assumes a constant force of infection over time and generally describes the results of cross-sectional IgG surveys well [3].

Binomial Simulation and Estimation Procedure

We considered 11 potential age ranges to test and 5 total survey sizes. We assumed that individuals were distributed uniformly across the age groups, i.e. for a total survey size of 2000 and an age range of 0-20 year olds; 96 individuals are tested in each year of age. We also considered 7 different transmission settings by varying the force of infection to give a true seroprevalence in 9-year olds (the target age group) which ranged from 10%-90% (P10 to P90). Finally, we looked at different test sensitivities and specificities (90% - 100%). Table S1 summaries the combinations tested.

For every combination of age range, survey size, transmission setting, and test sensitivity-specificity, we simulated 100 seroprevalence surveys. We assumed that the probability of an individual in age group $a$ being seropositive was binomially (Bin) distributed (2):

| $p_{a}\sim Bin\left(N_{a},z_{a}\right)$, |  | (2) |
| --- | --- | --- |

where $N_{a}$ is the number of individuals in age group $a$ and $z_{a}$ is the proportion of the age group seropositive given by equation (1). The sensitivity and specificity determines the probability that an individual in age group $a$ will test positive $\left(P\left(T_{a}^{+}\right)\right)$:

| $P\left(T_{a}^{+}\right)=p_{a}S_{e}+\left(1-p_{a}\right)\left(1-S_{p}\right)$ |  | (3) |
| --- | --- | --- |

Here $p_{a}$ is the probability an individual aged $a$ is seropositive (2) and $S_{e}$ and $S_{p}$ are the sensitivity and specificity of the IgG test, respectively. Thus, with a perfect test (100% sensitivity and specificity), $P\left(T_{a}^{+}\right)=p_{a}$. Fig shows the probability of testing positive at different sensitivity and specificities given that the baseline probability of being seropositive is 0.7.


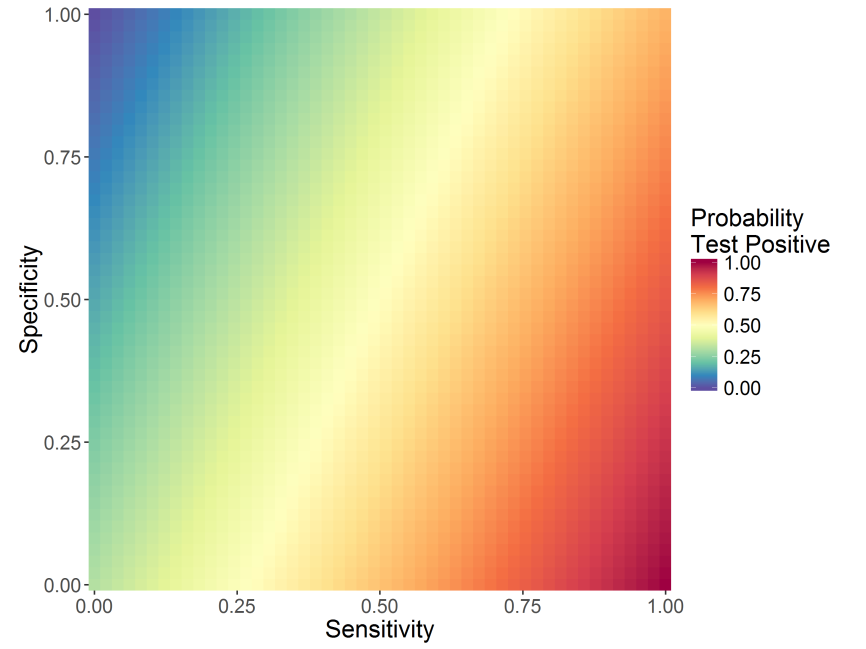


**Fig A**: Probability of 9 year olds testing positive [P(T_a_^+^)] at different test sensitivity and specificities. Baseline probability of being seropositive is 0.7 from a binomial distribution (p_9_ = 0.7).

The force of infection $\left(\lambda\right)$ was then re-estimated from each simulated dataset (100 estimates per parameter combination) using a Metropolis-Hasting Markov Chain Monte-Carlo (MH-MCMC) algorithm with a binomial likelihood (4) and uniform priors. The model log-likelihood is given by:

| $LnL=\sum_{a} X_{a}\log\left(P\left(T_{a}^{+}\right)\right)+\left(N_{a}-X_{a}\right)\log\left(1-P\left(T_{a}^{+}\right)\right)$ |  | (4) |
| --- | --- | --- |

Here $X_{a}$ is the number of individuals testing seropositive in age group $a$, $N_{a}$ is the total number of individuals tested in age group $a$, and $P\left(T_{a}^{+}\right)$ is the probability that an individual in age group $a$ tests positive as defined above (3).

The model was fitted using the R programming language (version 3.1.0) [4].

Table S1: Summary of different scenarios used for simulated seroprevalence surveys. Each variable is varied independently. The last three scenarios for the test sensitivity/specificity column represent reported performance of commercial tests.

| Age Range of survey (years) | Total Survey Size | Transmission Setting: % seroprevalence at age 9 (*λ*) | Test sensitivity / specificity |
| --- | --- | --- | --- |
| 0-20 |  |  |  |
| 5-20 |  | 10 (0.012) | 100% / 100% |
| 10-20 | 2000 | 30 (0.040) | 90% / 90% |
| 15-20 | 1500 | 50 (0.077) | 95% / 95% |
| 5-10 | 1000 | 60 (0.102) | 90% / 99% |
| 5-15 | 750 | 70 (0.134) | 99% / 90% |
| 5-18 | 500 | 80 (0.179) | 96.3% / 91.4% * |
| 9-12 |  | 90 (0.256) | 96% / 93% ^ |
| 9-15 |  |  | 98.8% / 99.2%¯ |
| 9-18 |  |  |  |

*PanBio IgG assay [16], ^Focus Diagnostics [17], ¯Standard Diagnostics Inc. [18].

We computed the mean, standard deviation and coefficient of variation (standard deviation divided by the mean) of the mean posterior estimates of the force of infection for each combination of age range, survey size, transmission setting, test sensitivity and test specificity.

**Results: Binomial**


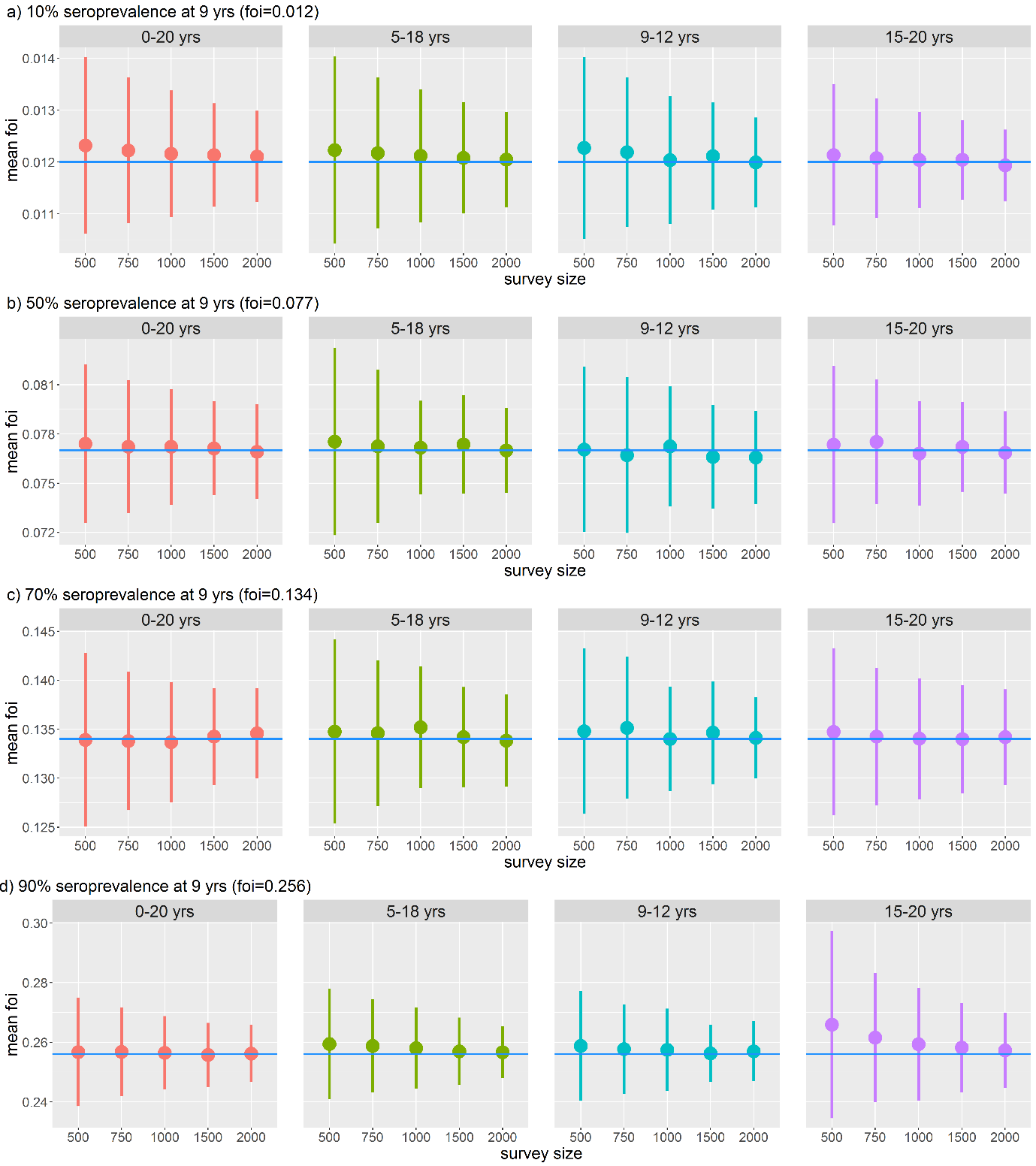


Fig B: Dengue force of infection (*λ*) re-estimated from different age ranges and survey sizes at different transmission settings

assuming a binomial distribution and a perfect test is used (100% sensitivity and specificity). A) Very low transmission setting (10% seroprevalence at 9 years), B) medium transmission setting (50% seroprevalence at 9 years), C) high transmission setting (70% seroprevalence at 9 years), and D) very high transmission setting (90% seroprevalence at 9 years). The point is the mean of the mean posterior estimates from 100 simulations and the line the standard deviation. The blue line shows the true value of *λ*.


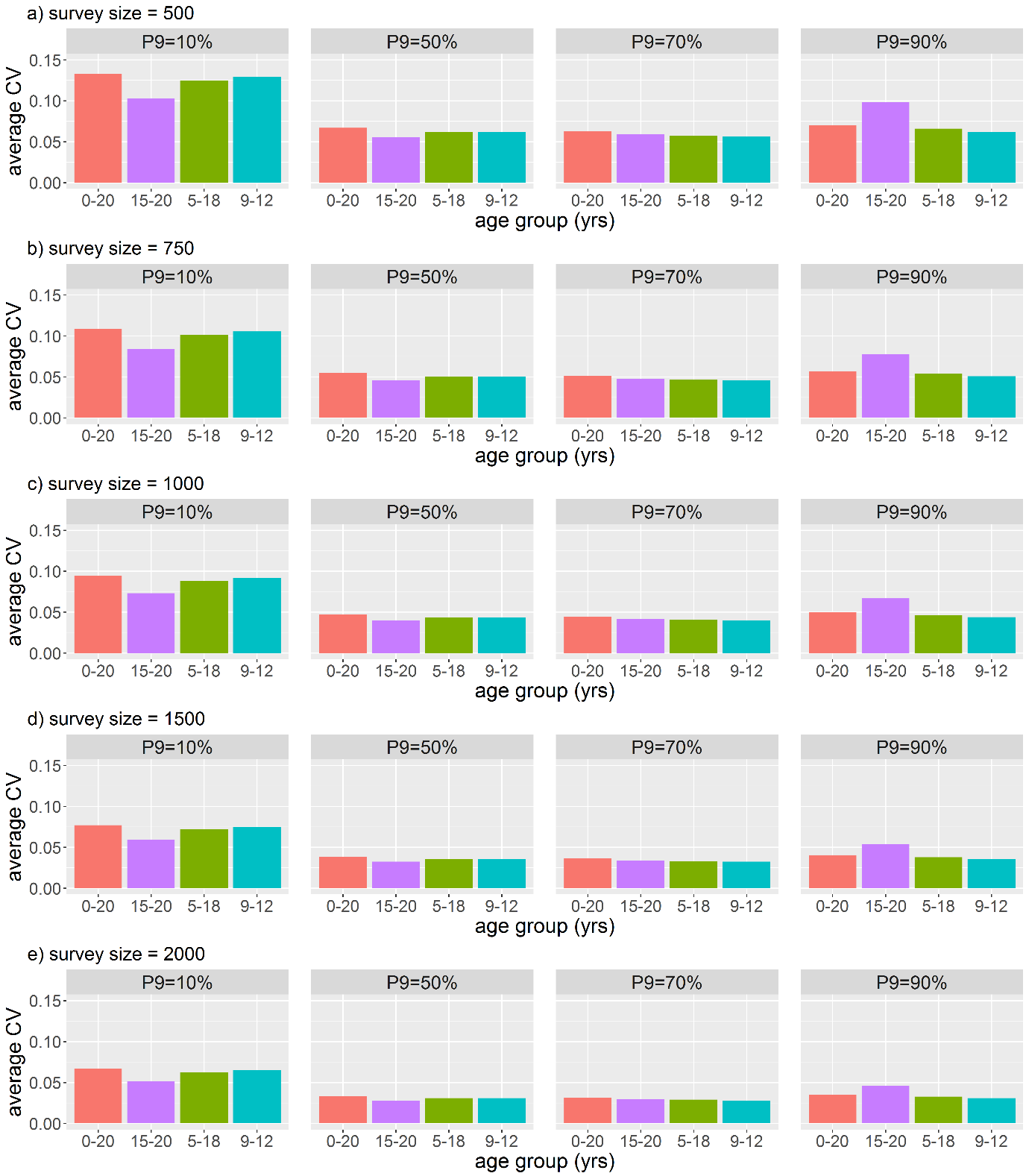
 Fig C: Average coefficient of variation (CV) for different age ranges sampled at different transmission settings

(P9 = expected seroprevalence at age 9). Results assume a binomial distribution and a perfect test (100% sensitivity and specificity) is used. Panels show total survey sizes of a) 500; b) 750; c) 1000; d) 1500; and e) 2000.


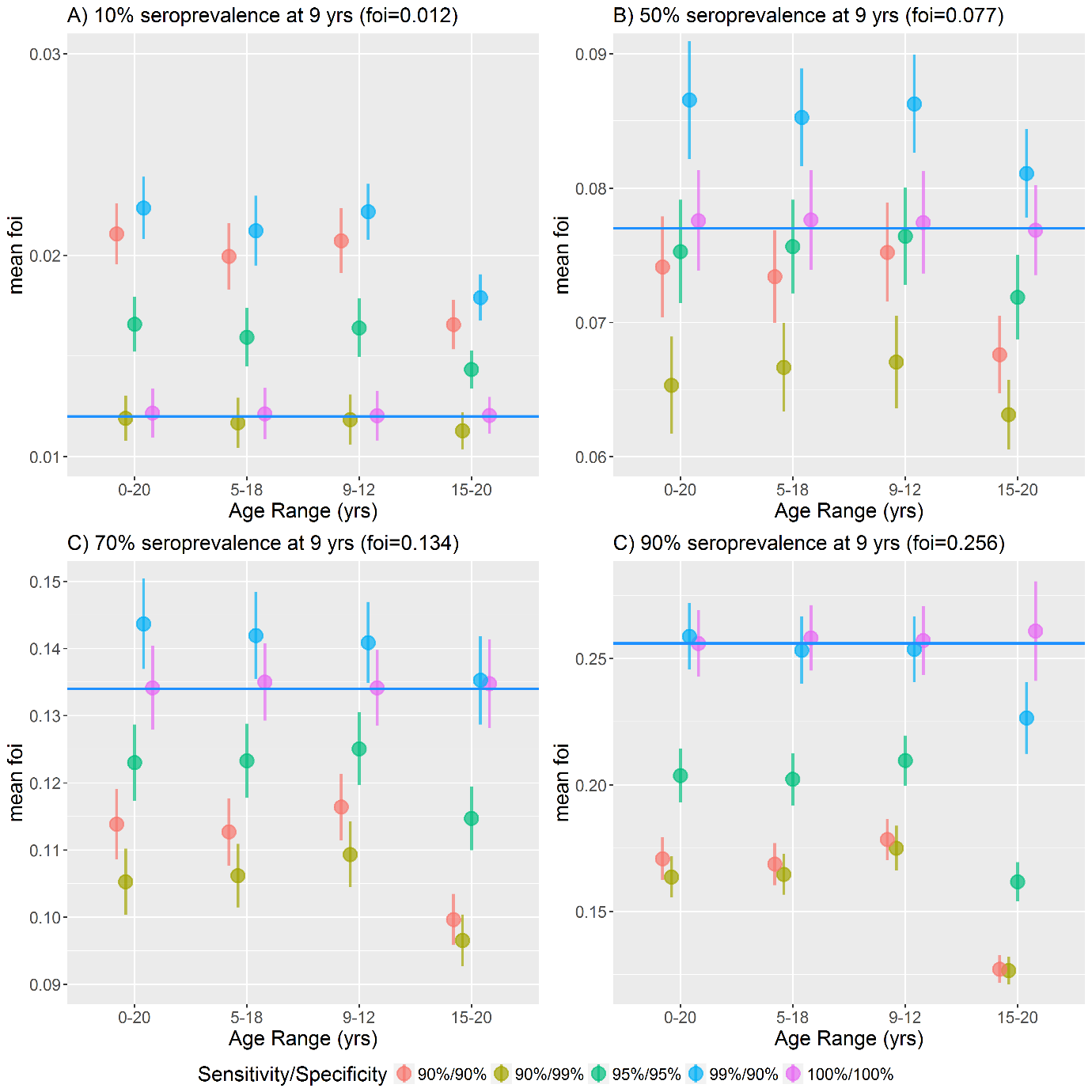
 Fig D: Dengue force of infection re-estimated at different transmission intensities, from a range of ages and test sensitivities and specificities, fixed survey size of 1000.

A) Very low transmission 10% seroprevalence at age 9, B) medium transmission 50% seroprevalence at age 9, C) high transmission 70% seroprevalence at age 9, D) very high transmission 90% seroprevalence at age 9. The point shows the mean of the 100 mean posterior distribution of the force of infection, the bar the standard deviation, and the horizontal blue line shows the true force of infection. Assumes a binomial distribution.


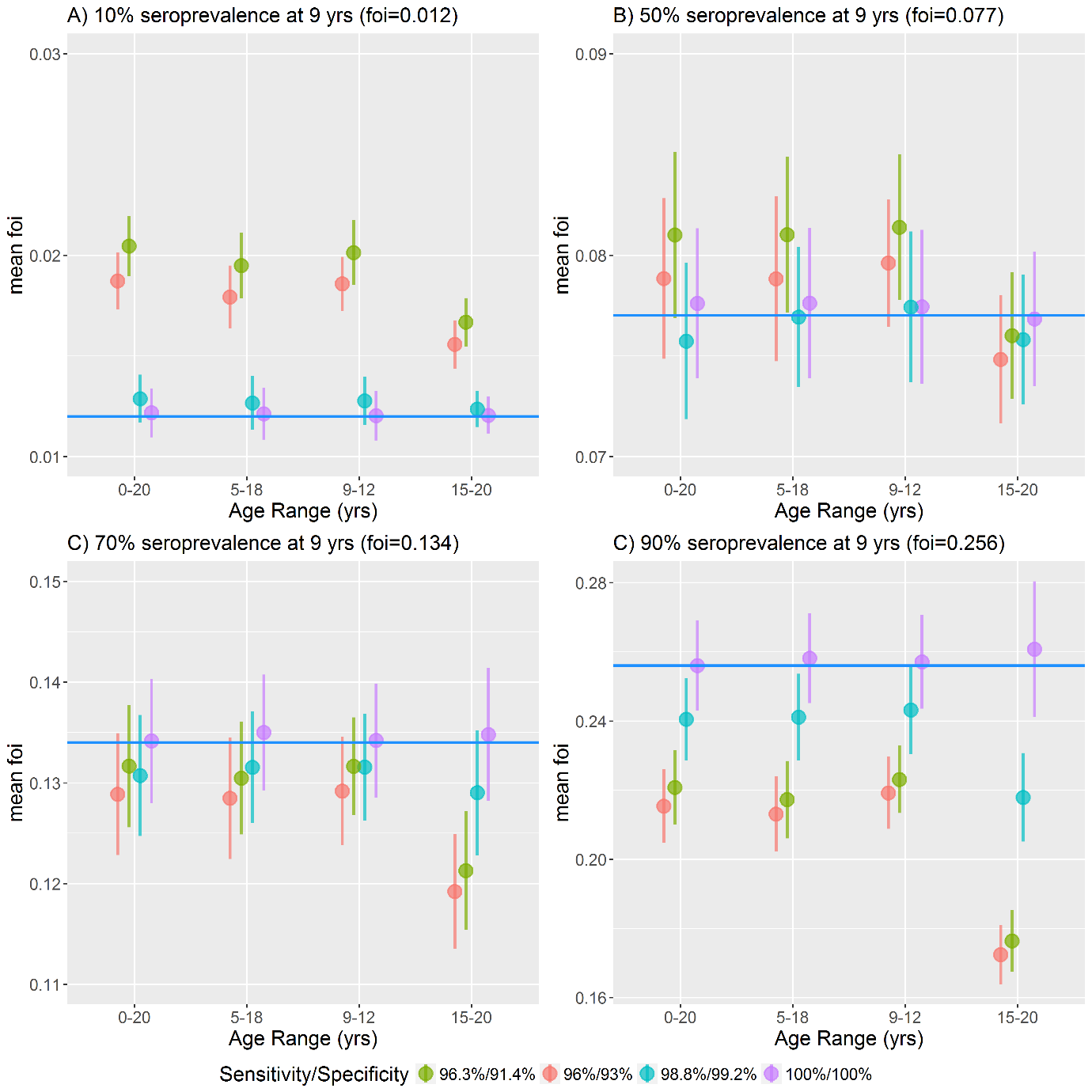
Fig E: Dengue force of infection re-estimated at different transmission intensities, from a range of ages and test sensitivities and specificities (commercial tests).

A) Very low transmission 10% seroprevalence at age 9, B) medium transmission 50% seroprevalence at age 9, C) high transmission 70% seroprevalence at age 9, D) very high transmission 90% seroprevalence at age 9. The point shows the mean of the 100 mean posterior distribution of the force of infection, the bar the standard deviation, and the horizontal blue line shows the true force of infection. Assumes a binomial distribution. Green = PanBio Indirect IgG [5], red = Focus Diagnostics IgG [6], blue = Standard Diagnostics IgG [7], purple = “perfect” assay.


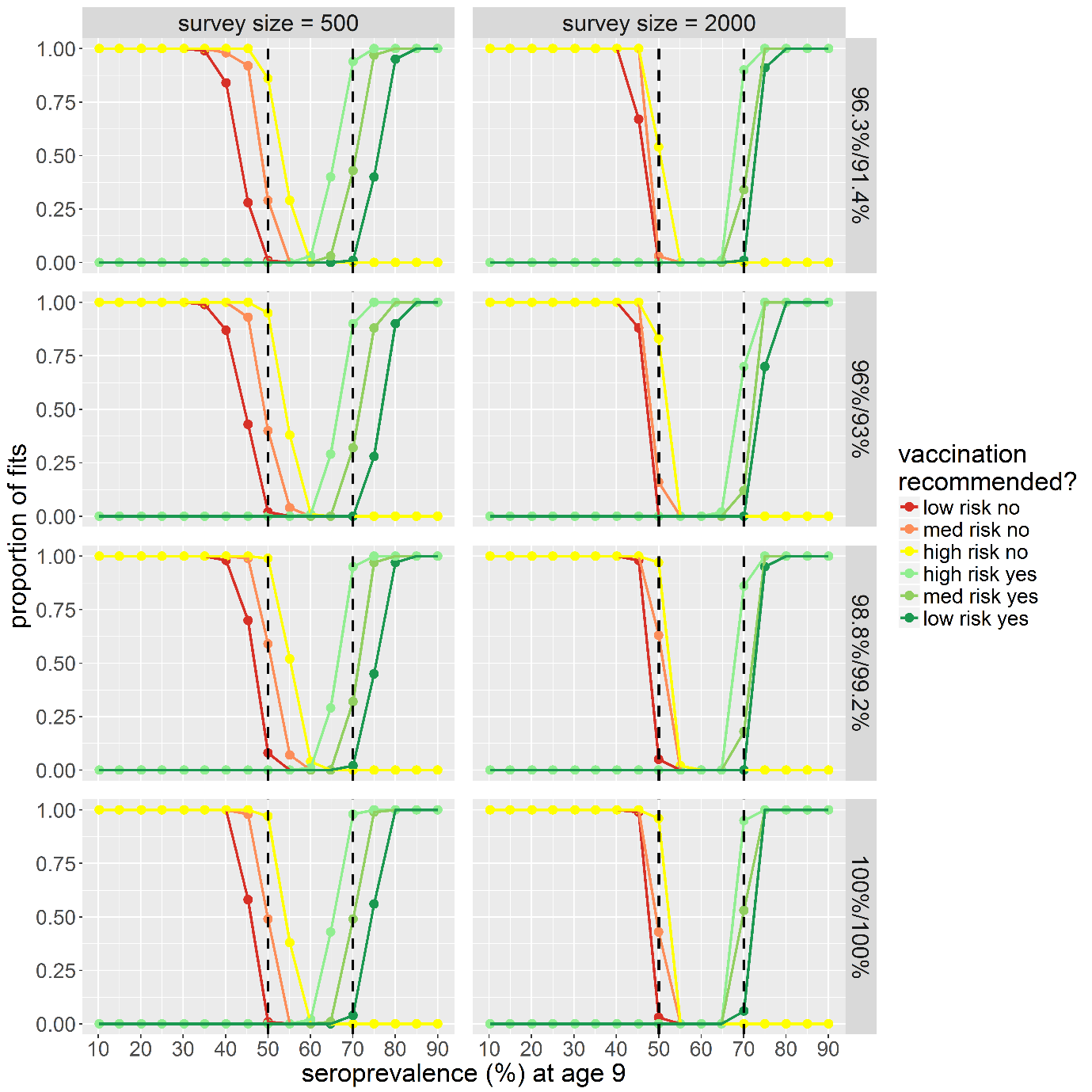


**Fig F**: Proportion of estimates which result in the correct vaccination recommendation for a range of seroprevalences in 9 year olds. Rows show results for different test sensitivities and specificities (Se%/Sp%). Columns for survey sizes of 500 and 2000, estimated from a fixed age range or 0-20 year olds. The black dashed lines represent seroprevalence thresholds of 50% and 70%. Red = vaccination is not recommended, green = vaccination is recommended. Low risk no = upper 95% CrI < 50%, med risk no = central estimate <50%, high risk no = upper 95% CrI < 50%, high risk yes = upper 95% CrI above 70%, med risk yes = central estimate >70%, low risk yes = lower 95% CrI >70%. CrI = credible interval. Sensitivity/Specificity (%): 96.3/91.4 = PanBio Indirect IgG [5], 96/93 = Focus Diagnostics IgG [6], 98.8/99.2 = Standard Diagnostics IgG [7]. Assumes a binomial distribution.

Additional Results: Beta-binomial model


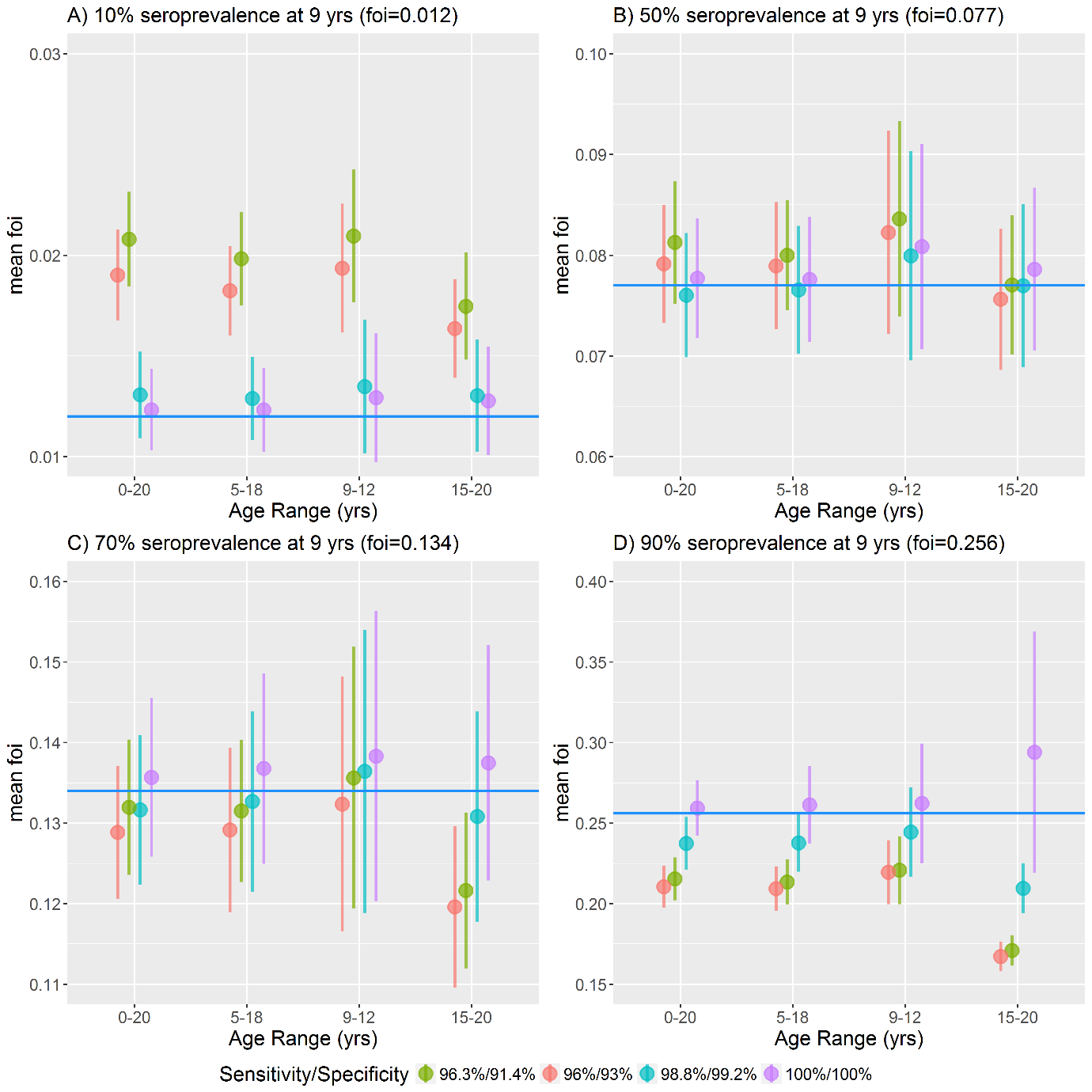


Fig G: Dengue force of infection re-estimated at different transmission intensities, from a range of ages and test sensitivities and specificities (commercial tests).

A) Very low transmission 10% seroprevalence at age 9, B) medium transmission 50% seroprevalence at age 9, C) high transmission 70% seroprevalence at age 9, D) very high transmission 90% seroprevalence at age 9. The point shows the mean of the 100 mean posterior distribution of the force of infection, the bar the standard deviation, and the horizontal blue line shows the true force of infection. Assumes a beta-binomial distribution. Green = PanBio Indirect IgG [5], red = Focus Diagnostics IgG [6], blue = Standard Diagnostics IgG [7], purple = “perfect” assay.


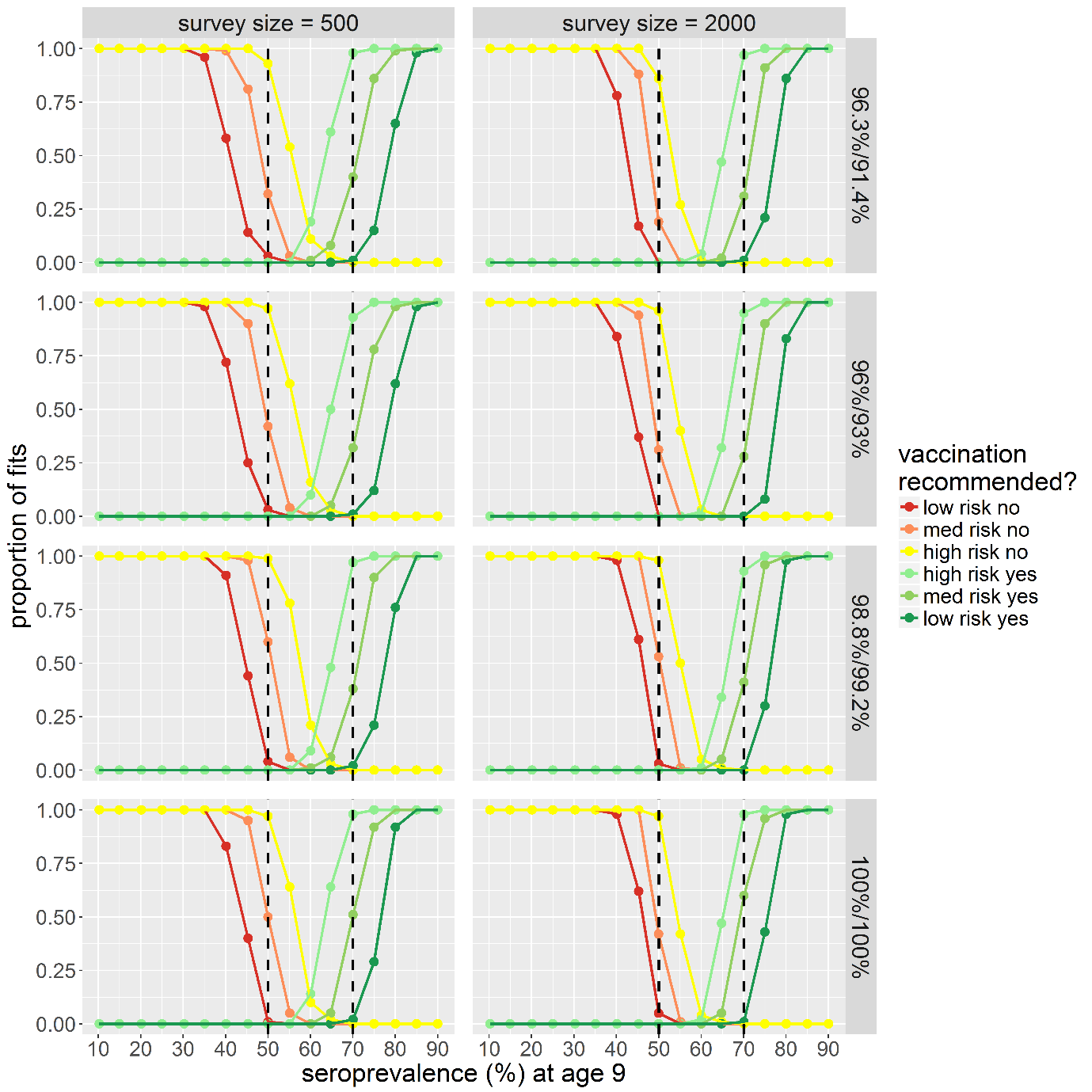


**Fig H:** Proportion of estimates which result in the correct vaccination recommendation for a range of seroprevalences in 9 year olds. Rows show results for different test sensitivities and specificities (Se%/Sp%). Columns for survey sizes of 500 and 2000, estimated from a fixed age range or 0-20 year olds. The black dashed lines represent seroprevalence thresholds of 50% and 70%. Red = vaccination is not recommended, green = vaccination is recommended. Low risk no = upper 95% CrI < 50%, med risk no = central estimate <50%, high risk no = upper 95% CrI < 50%, high risk yes = upper 95% CrI above 70%, med risk yes = central estimate >70%, low risk yes = lower 95% CrI >70%. CrI = credible interval. Sensitivity/Specificity (%): 96.3/91.4 = PanBio Indirect IgG [5], 96/93 = Focus Diagnostics IgG [6], 98.8/99.2 = Standard Diagnostics IgG [7]. Assumes a beta-binomial distribution.

Greater overdispersion

Here we assume greater overdispersion, which represents the underlying variation in transmission intensity within the sampled population, in the data where γ=15 (γ=25 in the main text). With greater overdispersion, it is increasingly difficult to accurately re-estimate the force of infection and the overestimation is more pronounced.


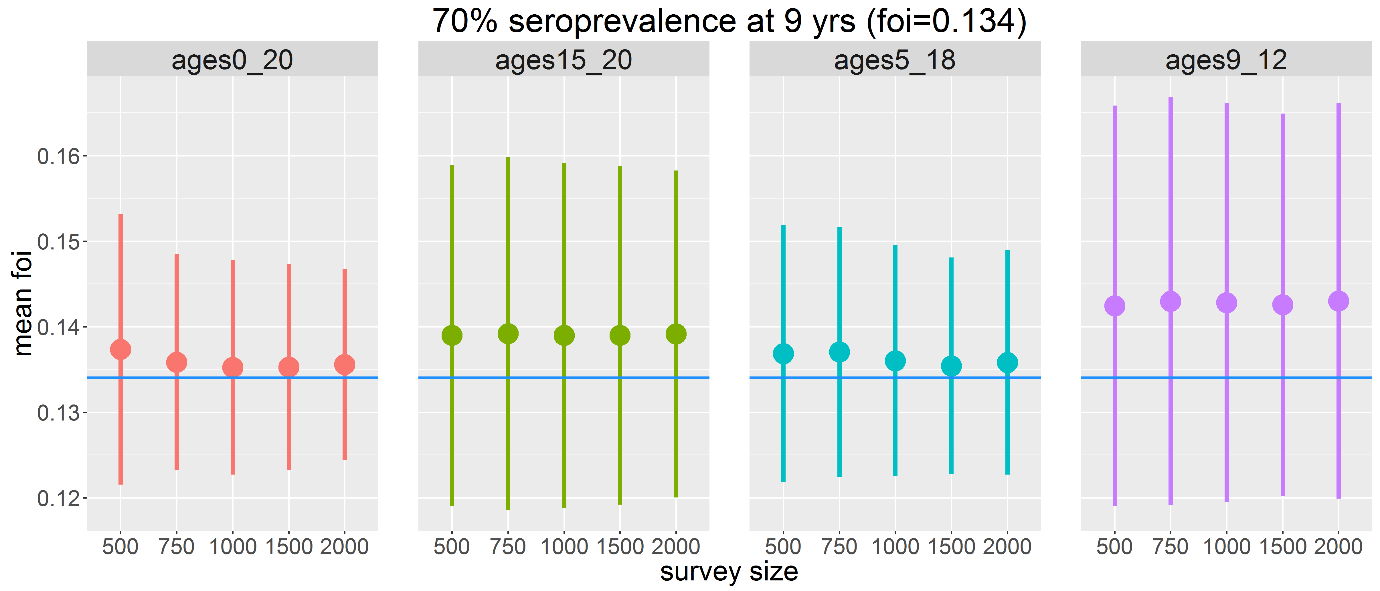


Fig I: Dengue force of infection (*λ*) re-estimated from different age ranges and survey sizes at high transmission setting (P9=70%) assuming a beta-binomial distribution with large overdispersion and a perfect test is used (100% sensitivity and specificity). The point is the mean of the mean posterior estimates from 100 simulations and the line the standard deviation. The blue line shows the true value of *λ*.

Difference in Precision – demonstrated using beta-binomial model

**
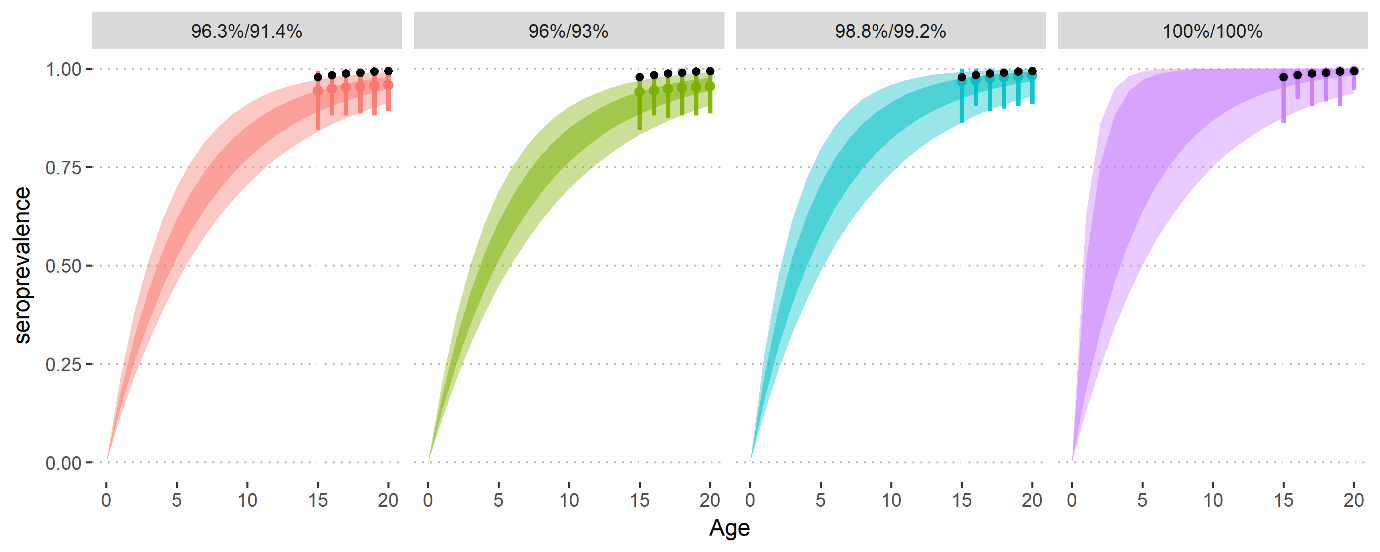
Fig J**: Range of simulated data and model fits for ages 15-20 at baseline seroprevalence of 90% in 9 year olds (P9=90%). The black points show the expected seroprevalence assuming this baseline transmission intensity, a perfect test, and no overdispersion. Each panel shows results for different test sensitivity and specificity. The coloured points and bars show the median value and range of the simulated data at each age. The dark shaded area the range of median posterior estimates of the model fit, and the light shaded area the minimum 2.5% credible interval and maximum 97.5% credible interval.

Comparisons of Root Mean Square Error: observed seroprevalence vs estimated seroprevalence

Due to the epidemic nature of dengue transmission and sampling variability, age-stratified seroprevalence surveys can show considerable fluctuations with age. Therefore, the observed seroprevalence in a single age group (e.g. 9 years) may not be representative of the true seroprevalence and transmission intensity of the target age group. However, by sampling from a wide age range, the information in other age groups can be leveraged to estimate the average transmission intensity of the population, and thus the seroprevalence in the target age group. In the figure below, we show the root mean square difference (rmsd) between the long-term average seroprevalence and a sample of seroprevalence observed in a single survey, and compare this to the rmsd between the long-term average seroprevalence and seroprevalence estimated using the force of infection estimated from an age-stratified serosurvey data. We can see that in every transmission setting and for all test sensitivity/specificity scenarios, the long-term average seroprevalence in 9 year olds is better assessed by estimating the force of infection from an age-stratified serosurvey.


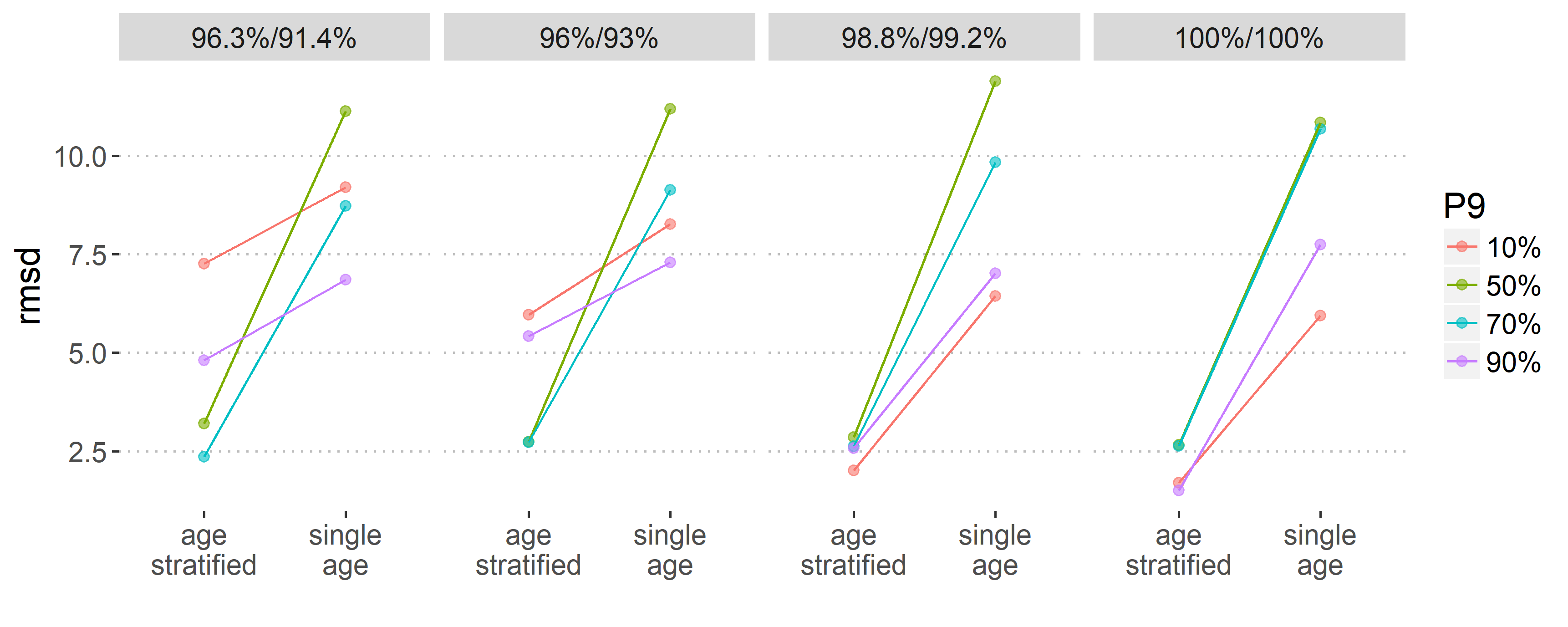


**Fig K:** Comparison of the root mean square difference (rmsd) between the long-term average seroprevalence at age 9 estimates obtained from age-stratified serosurveys (‘age-stratified’) and a single survey of seroprevalence in that age group (‘single age’). Each panel shows results for different test sensitivity and specificity. For the ‘age-stratified’ estimates, the force of infection was estimated from the full 0-20 year age distribution. A total survey size of 1000 was assumed. Different colours represent different underlying transmission intensities.
